# The Interplay between Fear Reactivity and Callous–Unemotional Traits Predicting Reactive and Proactive Aggression

**DOI:** 10.3390/children11040379

**Published:** 2024-03-22

**Authors:** Nicholas D. Thomson, Sophie L. Kjærvik, Victoria J. Blondell, Laura E. Hazlett

**Affiliations:** 1 Department of Psychology, Virginia Commonwealth University, Richmond, VA 23284, USA; 2 Department of Surgery, Virginia Commonwealth University, Richmond, VA 23298, USA; sophie.kjaervik@vcuhealth.org (S.L.K.); victoria.blondell@vcuhealth.org (V.J.B.); laura.hazlett@vcuhealth.org (L.E.H.)

**Keywords:** callous–unemotional traits, conduct disorder, skin conductance, fear, reactive aggression, proactive aggression

## Abstract

Research has indicated that youths with CU traits are fearless, and this fearlessness plays a bidirectional role in both the development of CU traits and engagement in aggressive behavior. However, research specifically testing the role of fear in the association between CU traits and aggression is scarce. The goal of the current study was to test if fear reactivity, both conscious (self-report) and automatic (skin conductance reactivity; SCR), moderated the association between CU traits and aggression subtypes (reactive and proactive aggression). Participants included 161 adolescents (*M_age_* = 15 years) diagnosed with conduct disorder. CU traits were assessed using the self-report Inventory of Callous–Unemotional Traits. Conscious and automatic fear reactivity were measured during a virtual reality rollercoaster using the Self-Assessment Manikin and skin conductance reactivity (SCR), respectively. Hierarchical regressions found that high fear reactivity on SCR moderated the link between CU traits and reactive aggression, while feeling more excited during fear induction moderated the link between CU traits and proactive aggression. Overall, a possible explanation of our divergent findings between conscious and automatic fear may be the difference between the instinctual biological response to threat versus the cognitive and emotional appraisal and experience of threat. Implications for intervention strategies targeting emotional recognition and regulation in reducing aggression in CD populations are discussed.

## 1. Introduction

Conduct disorder (CD) is among the most prevalent and debilitating psychiatric disorders emerging during childhood or adolescence [1,2,3]. CD has a worldwide prevalence of ~3.2% and is estimated to be responsible for 5.75 million years of healthy life lost [4]. Unfortunately, the impact of CD extends beyond the formative years, as it linked to persistent lifelong adjustment issues. These challenges encompass problems related to mental, legal, criminal, social, occupational, and physical health [5,6]. Exacerbating CD is a specifier called callous–unemotional (CU) traits. Studies show that 12–46% of youths with CD present with significant levels of CU traits [7,8,9,10,11]. Youths with CD and CU traits demonstrate the standard CD symptoms (e.g., persistently rule- and norm-breaking, defying authority figures across situations, and repeatedly and seriously infringing upon the rights of others). They also exhibit a lack of remorse or guilt, a callous disregard for others’ feelings, shallow or deficient emotional responses, and/or a lack of concern for their own performance [12]. Compared to youths with CD, youths with CD and high levels of CU traits are more likely to engage in severe and chronic violence and criminal behavior [13,14]. Thus, CU traits designate a subgroup of youths with CD who are at much greater risk of enduring maladjustments.

### 1.1. Fear and CU Traits

Prior research has suggested that adolescents exhibiting high levels of CU traits possess a temperament marked by fearlessness [15]. This has led researchers to extend the “low-fear hypothesis”, which is commonly associated with antisocial behavior more generally, to CU traits. Specifically, adolescents with elevated CU traits tend to report diminished subjective fear when recalling emotionally charged life events [16], score lower on self-report fear assessments [17], exhibit reduced physiological arousal to emotional stimuli [18], and demonstrate lower awareness of behaviors that elicit fear in others [19]. The low fear response has been attributed to deficits in emotional processing and the ability to see others’ perspectives [20]. Given that this fear deficit has been observed among preschoolers [21], fearlessness can exacerbate typical social development and play a bidirectional role in the development of CU traits [22]. The absence or dampened experience of fear among adolescents with elevated CU traits is thought to increase risky behaviors. For example, a child with high CU traits who has low levels of fear may be less deterred by the potential consequences of callous behavior (e.g., violence), such as being injured or punishment as a result of fighting. Therefore, fearlessness can strengthen the association between CU traits and specific types of aggressive behavior. However, this remains to be tested.

It is important to note that the low-fear hypothesis of CU traits is not without controversy [22,23,24,25]. A great deal of this criticism stems from the lack of consistency in defining and measuring fear [26], which may have inflated the relation between CU traits and fear [27]. The term “fear” has been vaguely used in research, often conflating two distinct processes of fear, conscious and automatic, both of which control the defensive response to threat [28]. Automatic fear is the nonconscious automatic threat detection and response process controlled by lower-level brain mechanisms, particularly within the limbic system, like the amygdala [28]. An automatic fear response is rapid and instinctual, occurring without the conscious appraisal of a threat. Automatic fear readies the body to manage the threat, such as increasing sympathetic nervous system (SNS) activity to support the fight or flight response. In contrast, conscious fear refers to the subjective experience and emotional feeling of being afraid. Conscious fear involves cognitive processes (e.g., appraisal, attention, and interpretation), awareness, and the higher brain regions, such as the cortex. Conscious fear is what people are typically aware of when they think or talk about being scared (e.g., self-report to fear induction). Ref. [28] suggested that, while both conscious and automatic fear are related, they are not the same construct—the automatic processes can occur without conscious fear, and conscious fear cannot be directly tied to the immediate automatic responses to a threat.

### 1.2. Fear, CU Traits, and Aggression

The distinction between conscious and automatic fear processing may be essential in understanding the relation between CU traits and aggression subtypes, such as reactive and proactive aggression. Proactive aggression is deliberate and planned behavior aimed at attaining a specific goal or advantage, often without emotional arousal, and typically viewed as a cold, calculated type of aggression [29,30,31]. In contrast, reactive aggression is an impulsive and reactive response to a perceived threat or provocation, characterized by an immediate defensive aggressive reaction [31,32,33]. Overall, proactive aggression is linked to the hypoarousal of the autonomic nervous system (ANS), while reactive aggression is associated with a hyperarousal to fear in youths with high CU traits [34,35]. Understanding the interplay between conscious and automatic fear reactivity may be essential in the association between CU traits and types of aggression, given the divergent mechanisms for reactive and proactive aggression—hyperarousal and hypoarousal, respectively. Although CU traits seem to be more related to proactive than reactive aggression [36,37,38], previous research has found that youths with high levels of CU traits exhibit both reactive and proactive aggression [39]. A reduced fear response may make youths high in CU traits more prone to engage in aggressive behavior, as they are less deterred by potential negative consequences. Thus, understanding how fear contributes to CU traits’ association with reactive and proactive aggression may be important for developing violence prevention strategies for youths with CD and elevated CU traits.

### 1.3. The Present Study

The goal of the present study is to examine the moderating role of fear reactivity, both conscious and automatic, in the association between CU traits and aggression subtypes. Based on the limited research using both measures of fear [35], and the dual-pathway model of CU traits [40], we expect reactive aggression to be related to high CU traits and high SNS reactivity (high automatic fear), and high CU traits and self-report emotional distress to fear induction (conscious fear). In contrast, we expect the hypoarousal of the SNS (low automatic fear) to serve as a moderator of the relation between high CU traits and high proactive aggression. Given that proactive aggression is described as “cold-blooded” and CU traits are associated with diminished emotional reactivity, we expect lower conscious fear to serve as a moderator of the relation between CU traits and proactive aggression.

## 2. Method

### 2.1. Participants

Participants were recruited from a large healthcare network in Virginia. All participants had a current conduct disorder diagnosis made by a licensed mental health professional. A total of 161 participants aged 12–17 (*M_age_* = 15.65, *SD* = 1.35) years old were included in this study. Most participants were male (70.8%) and identified as African American (53.4%), White (39.1%), mixed race (White and African American; 4.4%), or other (3.1%). A prior power analysis using G*Power 3.1 [41], with the f-test, linear multiple regression, fixed-model R^2^ increased, and a power of 0.80, assuming a medium effect, estimated that a sample size of at least 81 was needed.

### 2.2. Procedure

Participants with a current conduct disorder diagnosis were recruited from a large healthcare network in Virginia. Caregiver–youth dyads were contacted by email and phone, and were provided information about the study. Interested participants were invited into the lab. Prior to study participation, the youths provided assent and caregivers provided consent. Assent and consent were conducted in different and private rooms. The experiment took place within the PI’s virtual-reality psychophysiology lab, which is a designated room for psychophysiological data acquisition with ample space for VR experiments. Caregivers completed assessments about the child in a separate interview room. This project is part of a larger study looking at how fear reactivity contributes to the stability of CU traits (R01MH123535: PI Thomson). Youth participants received USD 200 for their participation in the larger study. In line with the prior research [35,42], the participants completed the vanilla baseline in VR (termed from hereon as the control condition) and then the fear induction (VR rollercoaster). Prior to the VR experiment, the VR headset was placed on the participant’s head, and the individual was prompted to describe their surroundings to ensure familiarity with the environment and verify their ability to perceive the display. To ensure there were no recording issues, the VR experience was cast onto a monitor observable by the research team.

Participants underwent a 3 min control condition during which they were asked to sit quietly and attempt to relax. The 3 min control condition included a quiet room with a fish tank in VR. The fear induction included a rollercoaster that lasted for about 3 min. After the fear induction, participants reported their emotional state using an adapted version of the Self-Assessment Manikin [43], which included emojis representing arousal, valence, and dominance.

### 2.3. Measures

Callous–Unemotional Traits. The Inventory of Callous–Unemotional Youth version (ICU-Y; ref. [44]) was developed to assess CU traits in youths. The scale includes 24-items rated on a Likert-scale (0 = Not at all true, 3 = Definitely true). The scale comprises both positively (e.g., “I do not show my emotions to others”) and negatively (e.g., “I am concerned about the feelings of others”) worded items; negatively worded items were reverse-scored, so higher scores signified higher levels of CU traits. The reliability and validity of the ICU has been validated in prior research [44]. The ICU yielded good internal consistency in the present study (*a* = 0.80).

Reactive–Proactive Aggression Questionnaire. The Reactive–Proactive Aggression Questionnaire (RPQ; ref. [31]) is a self-report measure with 23 items rated on a 3-point Likert scale (0 = Never, 2 = Often). The scale measures two types of aggression: reactive aggression (11 items; e.g., “Reacted angrily when provoked by others”) and proactive aggression (12 items; e.g., “Used force to obtain money or things form others”). Higher scores indicate more aggressive behavior. Each subscale yielded good internal consistency in the present study (reactive; *a* = 0.83; proactive; *a* = 0.80).

Automatic fear response. Skin conductance was recorded using two Ag–AgCl electrodermal conductance electrodes affixed to the index and middle phalanges of the non-dominant hand. Isotonic gel was applied before securing the Velcro adhesive collars. Data were recorded with Biopac MP160 with a BioNomadix module transmitter (MP160-BIOPAC Systems Inc., Goleta, CA, USA), and sampled at 1000 Hz. Offline analysis was conducted using Biopac’s Acknowledge 5.0 software. Skin conductance was recorded using a low-pass filter of 1 Hz and a gain of 5 µS/V. The waveform was smoothed at 1000 samples. Change scores were derived by subtracting the mean values of SCL in the control condition from the mean values in the fear condition (e.g., VR fear condition—VR control = change score), which aligns with the previous research [45,46]. Negative values indicated a decrease in SCL, whereas positive-change scores indicated an increase in SCL.

Conscious fear response. A modified version of the Self-Assessment Manikin [43] assessed the reactivity of arousal, valence, and dominance in the fear condition. The SAM is a nonverbal pictorial scale developed to examine feelings across conscious dimensions of emotions. The present study modified the SAM and used emojis to represent emotional reactivity. Participants reported how they felt after each condition on a 10-point scale. The valence scale spans from a smiley and happy manikin (1) to a frowning and unhappy manikin (10). The arousal scale extends from a calm and drowsy manikin (1) to an enthusiastic and alert manikin (10). The dominance scale spans from a diminutive and out-of-control manikin (1) to a towering and in-control manikin (10). In line with the prior research (see [35]), to estimate conscious fear reactivity, we computed change scores by subtracting the control condition means from the fear condition means (change score = fear condition − control condition). On the arousal scale, negative values signified more relaxation, while positive values signified more excitement. On the valence scale, negative values signified increased happiness, while positive values signified increased sadness. On the dominance scale, negative values signified decreased control, while positive values signified increased dominant and control.

### 2.4. Data Analytic Plan

First, we conducted correlations among the primary study variables to understand the association between CU traits, aggression subtypes, and fear reactivity. Next, we conducted a series of hierarchical multiple regressions to assess the relation between CU traits, fear reactivity, and aggression subtype. All models adhered to the same steps. In step 1, sex, age, CU traits, and fear reactivity (e.g., skin conductance reactivity) were included. In step 2, the interaction term between CU traits and the fear reactivity measure was added. Significant interaction terms were analyzed using a simple slopes analysis [47]. Collinearity diagnostic tests found both the tolerance values (>0.72) and variance inflation factor (<1.34) to be acceptable [48]. SCR, arousal, valence, dominance, and CU traits values were transformed to z-values for normalization [49,50]. Analyses were conducted in R version 2023.09.1+494 [51].

## 3. Results

### 3.1. Missing Data

Participants (*n* = 12) with partially missing data were excluded from the analyses.

### 3.2. Correlations among Main Study Variables

The correlation between proactive and reactive aggression was positive (*r* = 0.48, *p* < 0.001). CU traits were significantly related to proactive aggression (*r* = 0.21, *p* < 0.01), but not to reactive aggression (*r* = 0.14, *p* = 0.07). SCR was negatively associated with age and positively related to sex (*p* < 0.05). Feeling dominance during the rollercoaster was negatively associated with arousal and positively related to age (*ps* < 0.05). CU traits were not correlated with reactive aggression or fear reactivity scales (SCR, arousal, dominance, or valence). The correlations are displayed in Table 1.

### 3.3. CU Traits, Automatic Fear Reactivity, and Reactive and Proactive Aggression

**Figure 1:**
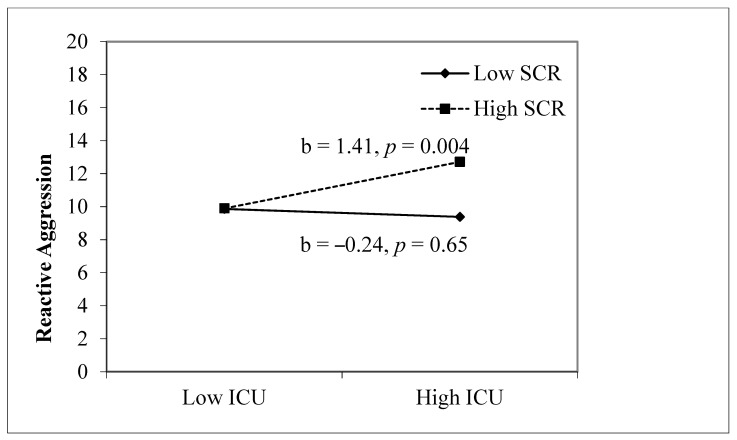
Interaction between CU traits and SCR predicting reactive aggression. Note. Sex and age are included as covariates. ICU, SCR, and age are z-scored.

Table 2 presents the findings for automatic fear reactivity (SCR) and CU traits predicting aggression. For reactive aggression, step 1, which included sex, age, CU traits, and SCR, was significant, *F*(4, 139) = 2.53, *p* = 0.044, and both SCR and sex were significant (*p* = 0.04 and *p* = 0.038, respectively). Step 2, which included the interaction term between CU traits and SCR was significant, *F*(5, 138) = 3.26, *p* = 0.008, and the interaction term was also significant (*p* = 0.017). Thus, the relation between CU traits and reactive aggression was moderated by a higher SCR. The simple slope analysis (see Figure 1) illustrates that reactive aggression is predicted by high SCR (+1 SD) and high CU traits (+1 SD), *b* = 1.41, *p* = 0.004. For proactive aggression, step 1 (*F*(4, 140) = 2.04, *p* = 0.092) and step 2 (*F*(5, 139) = 1.73, *p* = 0.13) were nonsignificant. Thus, the link between CU traits and proactive aggression was not moderated by SCR.

### 3.4. CU Traits, Conscious Fear Reactivity, and Reactive and Proactive Aggression

The results for Self-Assessment Manikin responses to the rollercoaster predicting reactive and proactive aggression are presented in Table 3.

**Figure 2:**
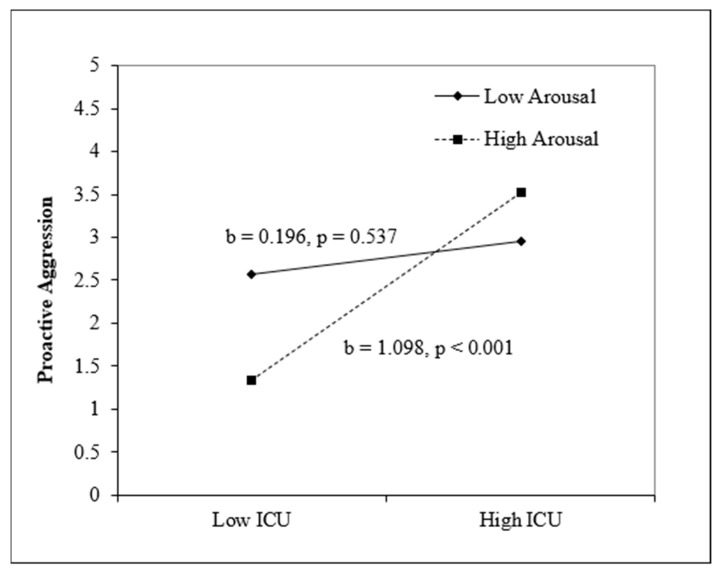
Interaction between CU and arousal predicting proactive aggression. Note. Sex and age are included as covariates.

Arousal. For reactive aggression, step 1, which included sex, age, CU traits, and arousal, was nonsignificant, *F*(4, 152) = 1.83, *p* = 0.13. Step 2 was also not significant, *F*(5, 151) = 1.79, *p* = 0.12. For proactive aggression, step 1, which included sex, age, CU traits, and arousal, was significant, *F*(4, 153) = 2.48, *p* = 0.046, and CU traits were positive and significant (*p* = 0.003). Step 2, which included the interaction term between CU traits and arousal, was also significant, *F*(5, 152) = 2.89, *p* = 0.016, and the interaction term between CU and arousal was significant (*p* = 0.040). The simple slope analysis (see Figure 2) illustrates that high CU traits and high arousal predicted proactive aggression, *b* = 0.13, *p* = 0.001.

Valence. For reactive aggression, step 1 was not significant, *F*(4, 152) = 1.65, *p* = 0.16. Step 2 was also not significant, *F*(5, 151) = 1.32, *p* = 0.26. For proactive aggression, step 1 was significant, *F*(4, 153) = 2.53, *p* = 0.043, and CU traits were positive and significant (*p* = 0.003). Step 2, which included the interaction terms between CU traits and valence was nonsignificant, *F*(5, 152) = 2.02, *p* = 0.079, and the interaction was nonsignificant (*p* = 0.85). Thus, valence response to fear did not affect the relation between CU traits and proactive aggression or reactive aggression.

Dominance. For reactive aggression, step 1, which included sex, age, CU traits, and dominance, was not significant, *F*(4, 152) = 1.36, *p* = 0.25. Step 2, was also nonsignificant, *F*(5, 151) = 1.17, *p* = 0.33. For proactive aggression, step 1 was significant, *F*(4, 153) = 2.53, *p* = 0.043, and CU traits were positive and significant (*p* = 0.003). Step 2 was nonsignificant, *F*(5, 152) = 2.01, *p* = 0.08. Thus, dominance response to the rollercoaster did not affect the relation between CU traits and proactive aggression.

## 4. Discussion

The association between CU traits and fear has been extensively debated, particularly under the guise of testing the low-fear hypothesis and the dual-pathway model of CU traits. While the relation between fear and CU traits has been tested, the role that fear plays in CU traits-related aggression is not well-understood. The present study found that fear reactivity, both conscious and automatic, played a unique role in the interplay between CU traits and aggression subtypes. In line with past research, we found CU traits were not associated with reactive aggression [36,37,38], but greater automatic fear reactivity (higher SCR) was linked to reactive aggression [35]. Furthermore, greater automatic fear reactivity (higher SCR) moderated the link between high CU traits and high reactive aggression. This finding was somewhat expected, as hyperarousal to fear is linked to reactive aggression. This resonates with the frustration–aggression hypothesis [52] and prior research demonstrating that a higher SCR is related to reactive aggression in response to stress [53]. Therefore, youths with CU traits who have a heightened sensitivity to threat engage in reactive aggression. While speculative, this finding may be driven by adverse childhood experiences, which are known to contribute to the development of hypersensitivity to threat [27]. What was not expected was that conscious fear reactivity did not affect the association between CU traits and reactive aggression. Prior research found that reactive aggression in a normative college sample was associated with a higher automatic fear response and feeling less in control during fear induction (greater conscious fear reactivity [35]). A possible explanation for our divergent findings between conscious and automatic fear is the difference between the instinctual biological response to threat versus the cognitive and emotional appraisal and experience of threat. As [28] states, these two processes occur independently; therefore, these functioning processes with CU traits and reactive aggression are interesting.

Automatic fear responses are unconscious and involve rapid physiological reactions to threats, typically mediated by the amygdala and related subcortical structures. These responses are thought to be relatively hardwired and occur without conscious deliberation [28]. This suggests that the relation between CU traits and reactive aggression is when youth with CU traits have hyperactive automatic threat processes. This may explain the mixed findings of some research linking CU traits to reactive aggression, while others do not [36,37,38]. This may suggest that CU trait-related reactive aggression is more automatic and driven by a physiological response than a conscious response to a threat.

CU traits were linked to proactive aggression, and conscious fear moderated this association. Specifically, high CU traits and greater feelings of excitement during fear induction were related to proactive aggression. This suggests that, despite being characteristically unemotional, youths with high CU traits experience positive effects in response to threat, which can explain the propensity for risk-taking associated with CU traits. While proactive aggression is thought to be driven by unemotionality or “cold-blooded” motivations, the present finding extends this and suggests that youths high in CU traits who are excited by a threat (i.e., fear induction) are more likely to engage in goal-directed and predatory aggression. This excitement, thrill, or rush associated with fear-inducing situations can reinforce risky behaviors, including proactive aggression, especially if they anticipate excitement as an outcome (which is a proactive motivation in itself). Indeed, prior research among youths found that CU traits were associated with the coactivation of the ANS, which may result in a more enjoyable experience during thrilling experiences, suggesting that youths with CU traits may be better able to manage threatening situations [27]. Collectively, this finding may suggest that the act of planned aggression can be enticing enough to spur youths with CU traits to pursue it. Notably, prior research has found that interpreting fear-inducing stimuli as exciting strengthens the relation between psychopathy and risk-taking [54], a concept known as the fear-enjoyment hypothesis [55]. Surprisingly, dampened automatic fear reactivity did not moderate the association between CU traits and proactive aggression. Prior research has found that the concurrent inhibition of the ANS (low SNS and PNS reactivity) is connected to proactive aggression [35], and low autonomic activity (e.g., heart rate) is linked to proactive aggression [56,57]. This may suggest that low ANS activity serves as a risk factor for proactive aggression among normative samples for aggression, but not for youths with CD and elevated CU traits. We encourage future research to investigate the role of ANS in different populations.

The results carry implications for prevention and intervention strategies. For instance, initiatives aimed at preventing aggression may benefit from targeting processes preceding aggressive behavior, including SCR and fear responses. Early intervention to foster positive development has the potential to decrease the incidence of conduct disorders and CU traits, with early interventions proving effective [58]. Additionally, interventions geared toward enhancing emotion regulation skills have the potential to decrease reactive aggression [59], whereas interventions that foster alternative goal-setting skills (e.g., cognitive behavioral therapy; [59]) or on the thrill-seeking aspect of aggression (e.g., risky behavior-reduction skills training) can decrease proactive aggression. Furthermore, the finding that excitement influences proactive aggression can enhance the development of more effective risk assessment tools. The effect of fear-induced arousal on the CU–aggression link can offer pathways for more precise and effective prevention and intervention efforts. Unfortunately, we did not explore the role of other psychosocial factors, such as childhood trauma, which could be relevant to our finding on reactive aggression, as trauma exposure is associated with greater threat sensitivity and CU traits [39,60].

### Limitations

The current paper should be interpreted with several limitations in mind. First, we included a VR rollercoaster, which is often an item used in fear measures (e.g., fear survey schedule). However, rollercoasters can map more onto thrill or adventure-seeking behavior rather than fear. Therefore, while the focus of this paper is on fear, future research is needed to explore immersive fear and thrilling stimuli to tease this association. Second, we did not have sufficient power to test for sex differences, which is important because male and female fear reactivity is differently related to aggression subtypes (see [35]). Thirdly, we did not account for the type of treatment youths were involved in (e.g., therapy modality or psychotropic), which could have impacted individual differences in emotion regulation. While the present study is novel in using two reports of fear, both conscious and automatic, we do not include a measure of the parasympathetic nervous system. The interaction between the SNS and PNS is predictive of both aggression subtypes and CU traits, so this is an important line of inquiry for future research. Lastly, the present study is cross-sectional, so the results are not able to determine if CU traits and fear reactivity predict aggression over time. Nevertheless, this study is a first for exploring the roles of automatic and conscious fear reactivity as moderators in the CU–aggression link among youths with CD.

## 5. Conclusions

The study findings extend our understanding of CU traits and reactive and proactive aggression. It supports the connection between hyperarousal to fear and reactive aggression. The intricate relationship is further clarified, with the revelation that the link between CU traits and reactive aggression is moderated by high fear reactivity on SCR. In addition, the findings lend support to the fear-enjoyment hypothesis, demonstrating that the link between CU traits and proactive aggression is moderated by increased excitement during fear induction. Overall, these findings contribute to our understanding of the complex interplay between fear reactivity, CU traits, and subtypes of aggression.

## Figures and Tables

**Table 1:** Correlations between main study variables.

	1	2	3	4	5	6	7	8	9
ICU	-								
2. Reactive	0.14	-							
3. Proactive	0.21 *	0.48 **	-						
4. SCR	−0.15	0.10	−0.05	−					
5. Arousal	0.07	0.09	−0.03	0.09	−				
6. Valence	−0.12	0.07	−0.06	0.10	−0.13	−			
7. Dominance	−0.04	−0.003	0.03	−0.004	−0.16 *	−0.14	−		
8. Age	0.06	0.09	−0.04	−0.17 *	0.04	−0.04	0.18 *	−	
9. Sex ^a^	0.10	−0.10	−0.07	0.19 *	0.11	−0.06	−0.08	−0.02	-
Mean	23.08	9.43	1.98	0.82	3.22	−0.49	−1.20	15.65	0.71
SD	8.67	4.59	2.73	1.70	3.09	2.60	3.96	1.35	0.46

Note. *p* < 0.05 *, *p* < 0.001 **. ^a^ Spearman’s correlation.

**Table 2:** SCR as a moderator of reactive and proactive aggression on ICU traits.

	Reactive Aggression				Proactive Aggression			
	*B*	SD	β	R^2^	*B*	SD	β	R^2^
Step 1				0.04 *				0.03
Sex	−1.70	0.81	−0.18 *		−0.02	0.49	−0.003	
Age	0.35	0.28	0.10		−0.14	0.17	−0.07	
CU	0.66	0.38	0.14		0.60	0.22	0.22 **	
SCR	0.79	0.39	0.17 *		−0.05	0.23	−0.02	
Step 2				0.07 **				0.02
Sex	−1.76	0.80	−0.18 *		−0.02	0.49	−0.003	
Age	0.37	0.38	0.08		−0.16	0.17	−0.08	
CU	0.59	0.38	0.13		0.60	0.22	0.20 **	
SCR	0.84	0.38	0.19 *		−0.04	0.23	−0.16	
CU × SCR	0.83	0.34	0.20 *		0.14	0.20	0.15	

Note. *n* = 144, *p* < 0.05 *, *p* < 0.01 **. Sex = male (0) and female (1).

**Table 3:** Conscious fear reactivity as moderator of reactive and proactive aggression on ICU traits.

	Reactive Aggression				Proactive Aggression			
	*B*	SD	β	R^2^	*B*	SD	β	R^2^
**Arousal**								
Step 1				0.02				0.04 *
Sex	−1.11	0.79	−0.11		0.03	0.46	0.006	
Age	0.29	0.27	0.09		−0.13	0.15	−0.07	
CU	0.56	0.37	0.12		0.65	0.21	0.24 **	
Arousal	0.49	0.36	0.11		−0.03	0.21	−0.01	
Step 2				0.02				0.06 *
Sex	−1.08	0.79	−0.11		0.06	0.46	0.01	
Age	0.26	0.27	0.08		−0.16	0.15	−0.08	
ICU	0.56	0.37	0.12		0.65	0.21	0.24 **	
Arousal	0.51	0.36	0.11		−0.02	0.21	−0.006	
CU × Arousal	0.48	0.38	0.10		0.45	0.22	0.16 *	
**Valence**								
Step 1				0.02				0.04 *
Sex	−0.97	0.79	−0.10		0.02	0.46	0.003	
Age	0.31	0.27	0.09		−0.13	0.15	−0.07	
CU	0.64	0.38	0.14		0.64	0.21	0.24 **	
Valence	0.39	0.36	0.09		−0.10	0.21	−0.04	
Step 2				0.01				0.03
Sex	−0.98	0.80	−0.10		0.03	0.47	0.005	
Age	0.31	0.27	0.09		−0.13	0.16	−0.07	
CU	0.64	0.38	0.14		0.64	0.21	0.24 **	
Valence	0.40	0.37	0.09		−0.10	0.21	−0.04	
CU × Valence	0.03	0.34	0.008		−0.03	0.19	−0.01	
**Dominance**								
Step 1				0.009				0.04 *
Sex	−1.02	0.79	−0.10		0.03	0.46	0.006	
Age	0.31	0.27	0.09		−0.14	0.16	−0.07	
CU	0.58	0.37	0.12		0.65	0.21	0.24 **	
Dominance	−0.07	0.37	−0.02		0.09	0.21	0.04	
Step 2				0.005				0.03
Sex	−1.02	0.79	−0.10		0.03	0.46	0.006	
Age	0.33	0.28	0.10		−0.14	0.16	−0.07	
CU	0.56	0.38	0.12		0.65	0.21	0.24 **	
Dominance	−0.04	0.37	−0.009		0.09	0.21	0.04	
ICU × Dominance	0.26	0.40	0.05		−0.002	0.22	−0.0006	

Note. *n* = 162, *p* < 0.05 *, *p* < 0.01 **.

## Data Availability

The data presented in this study may be made available upon request from the corresponding authors. The data are not publicly available due to the potential for personal identification of participants in the present sensitive population.
